# Identification of molecular mechanisms causing skin lesions of cutaneous leishmaniasis using weighted gene coexpression network analysis (WGCNA)

**DOI:** 10.1038/s41598-023-35868-0

**Published:** 2023-06-17

**Authors:** Kavoos Momeni, Saeid Ghorbian, Ehsan Ahmadpour, Rasoul Sharifi

**Affiliations:** 1 Department of Molecular Genetics, Ahar Branch, Islamic Azad University, Ahar, Iran; 2 Infectious and Tropical Disease Research Center, Tabriz University of Medical Sciences, Tabriz, Iran; 3 Department of Biology, Faculty of Basic Science, Ahar Branch, Islamic Azad University, Ahar, Iran

**Keywords:** Computational biology and bioinformatics, Genetics, Molecular biology, Systems biology, Biomarkers

## Abstract

Leishmaniasis is an infectious disease, caused by a protozoan parasite. Its most common form is cutaneous leishmaniasis, which leaves scars on exposed body parts from bites by infected female phlebotomine sandflies. Approximately 50% of cases of cutaneous leishmaniasis fail to respond to standard treatments, creating slow-healing wounds which cause permanent scars on the skin. We performed a joint bioinformatics analysis to identify differentially expressed genes (DEGs) in healthy skin biopsies and Leishmania cutaneous wounds. DEGs and WGCNA modules were analyzed based on the Gene Ontology function, and the Cytoscape software. Among almost 16,600 genes that had significant expression changes on the skin surrounding Leishmania wounds, WGCNA determined that one of the modules, with 456 genes, has the strongest correlation with the size of the wounds. Functional enrichment analysis indicated that this module includes three gene groups with significant expression changes. These produce tissue-damaging cytokines or disrupt the production and activation of collagen, fibrin proteins, and the extracellular matrix, causing skin wounds or preventing them from healing. The hub genes of these groups are *OAS1, SERPINH1,* and *FBLN1* respectively. This information can provide new ways to deal with unwanted and harmful effects of cutaneous leishmaniasis.

## Introduction

Leishmaniasis is caused by protozoan Leishmania parasites from over 20 *Leishmania* species that are transmitted via the bite of over 90 sandflies species of the infected female ones.

Cutaneous leishmaniasis (CL) is the most common form that causes skin lesions, mainly ulcers, on exposed parts of the body, which although not lethal, leave life-long scars. It is estimated that 12 million people are currently infected and around 2 million infections occur yearly^1^.

Cutaneous leishmaniasis is an endemic infection in the Middle East and South America. The most common strains of Anthropologic Cutaneous Leishmaniosis (ACL) in said regions are Leishmania Tropica and Leishmania Brasiliensis respectively^2^.

Diagnosis and treatment of cutaneous leishmaniasis have a few bottlenecks. The gold standard for its diagnosis is the observation of parasitic protozoa in skin wound samples. Even though serological and molecular tests can diagnose the infection with a high degree of certainty, none can predict the disease's treatment course and suggest appropriate medicine^1^. Antimony and Glucantime are the most common drugs for CL, but some patients do not respond to them appropriately, which prolongs the course of treatment, exposing the side effects of the drugs and the wound spreads^1,2^.

Next-Generation Sequencing (NGS) and high-throughput sequencing techniques have made it possible to diagnose and treat the majority of infections in new ways. Analyzing transcriptomic data from infected cells guides us to Identify the cause of the disease and determine the most appropriate treatment^3^.

Sequencing infected cells' mRNA molecules and determining the expression level of various genes made a revolution in diagnosing the causes of diseases. This way, we can identify shifts in the quantity and the type of biomolecules within cells and comprehend the molecular basis for clinical changes^4^. Several papers indicate that the over-activation of a patient's immune system plays a significant role in the pathogenesis of leishmaniasis^5^, but the alteration of the proteins that are effective in the maintenance of skin and wounds' healing process has rarely been studied.

Weighted Gene Co-expression Network Analysis (WGCNA) is one of the best Genomic analysis methods, focusing on a group of genes rather than a single gene to limit bias. WGCNA does not require cut-off criteria and may retrieve important information, which in any other case may be ignored. WGCNA, by converting a gene co-expression similarity matrix into a network connection strength matrix, can create gene co-expression modules and summarize them into module eigengene^6^.

Since our knowledge of the pathogenesis factors which cause Leishmania ulcers is limited, it is difficult to choose an appropriate and efficient treatment method. Although a lot of research has been done on this subject, the molecular and genetic causes of skin ulcers are still not completely clear. In this research, we have tried to investigate the cause of skin ulcers in cutaneous leishmaniasis by bioinformatics analysis of the data available in the databases. Here, Using Differentially Expressed Genes (DEGs) and weighted gene coexpression network analysis (WGCNA), we report modules that might provide potential biomarkers and therapeutic targets for more accurate diagnosis and treatment of wounds caused by cutaneous leishmaniasis (CL).

## Methodology

### Data information

We downloaded all the data used in this research from National Center for Biotechnology Information Gene Expression Omnibus (https://www.ncbi.nlm.nih.gov/geo/). We used the data set GSE127831^7^ to construct a co-expression network by Differential Gene Expression analysis (DGEs) and Weighted Gene Co-expression Network Analysis (WGCNA). The Data was obtained from RNA sequencing the skin cells of 21 patients with cutaneous leishmaniasis, and seven healthy people. The genes are tested individually for expression differences between conditions. Using the results of these analyses we can accurately identify the main damaging factor of leishmaniasis and suggest more effective methods for treatment. This would rely on the gene expression profile of each sample.

The samples (n = 28) were divided into two groups, the Healthy skin biopsy (n = 7) and the Leishmania cutaneous wound biopsy (n = 21). The raw data and study design files were imported into the R software^8^. A variety of R packages were used for this analysis. All graphics and data wrangling was created using the "Tidyverse" suite of packages^9^. All packages used are available from the Comprehensive R Archive Network (CRAN)^8^, Bioconductor.org^10^, or Github^11^.

Filtering is carried out to remove lowly expressed genes. Genes with less than 1 Count Per Million (CPM) in at least seven samples were filtered out. Therefore, the number of genes decreased from 34,935 to 16,665. The data were normalized using the TMM method^12^ in EdgeR^13^. We produced a table for the filtered and normalized data (Table S1). The table includes the expression data for 16,665 genes. The scattering curve and median of filtered and normalized data (Table S1) for each sample were plotted (Fig. S1). Based on the pattern of the expression of genes in each sample we could draw a cluster dendrogram that can distinguish between healthy and patient samples (Fig. S2). The data were analyzed using “Principal Component Analysis (PCA)”^14^ and the genes were classified into several clusters. The percentage of the genes in the first two major clusters were 55.7% and 6.9% respectively, and the classification curves of the samples based on clusters one and two are shown in Fig. S3.

### Differential gene expression analysis (DGEs)

Differential gene expression analysis involves taking the normalized read count data (Table S1) and performing statistical analysis to uncover quantitative differences in the expression levels between each experimental group. Analyzing differential expression can be done in a variety of ways. Appropriate analysis methods should be chosen based on the experimental design.

After setting up the design matrix based on the "Disease" trial, we could model the mean–variance relationship using the VOOM function from the Limma package^15^, prepare a "Top Hits" table (Table S2), and plot a volcano plot of the data (Fig. S4). Then we separated the significant differentially expressed genes, (adj. P value > 0.05 and LogFC > 0.8) from the "Top Hits". This way, the number of selected genes was limited to 6096 (Fig. S5) (Table S3).

**Figure 1:**
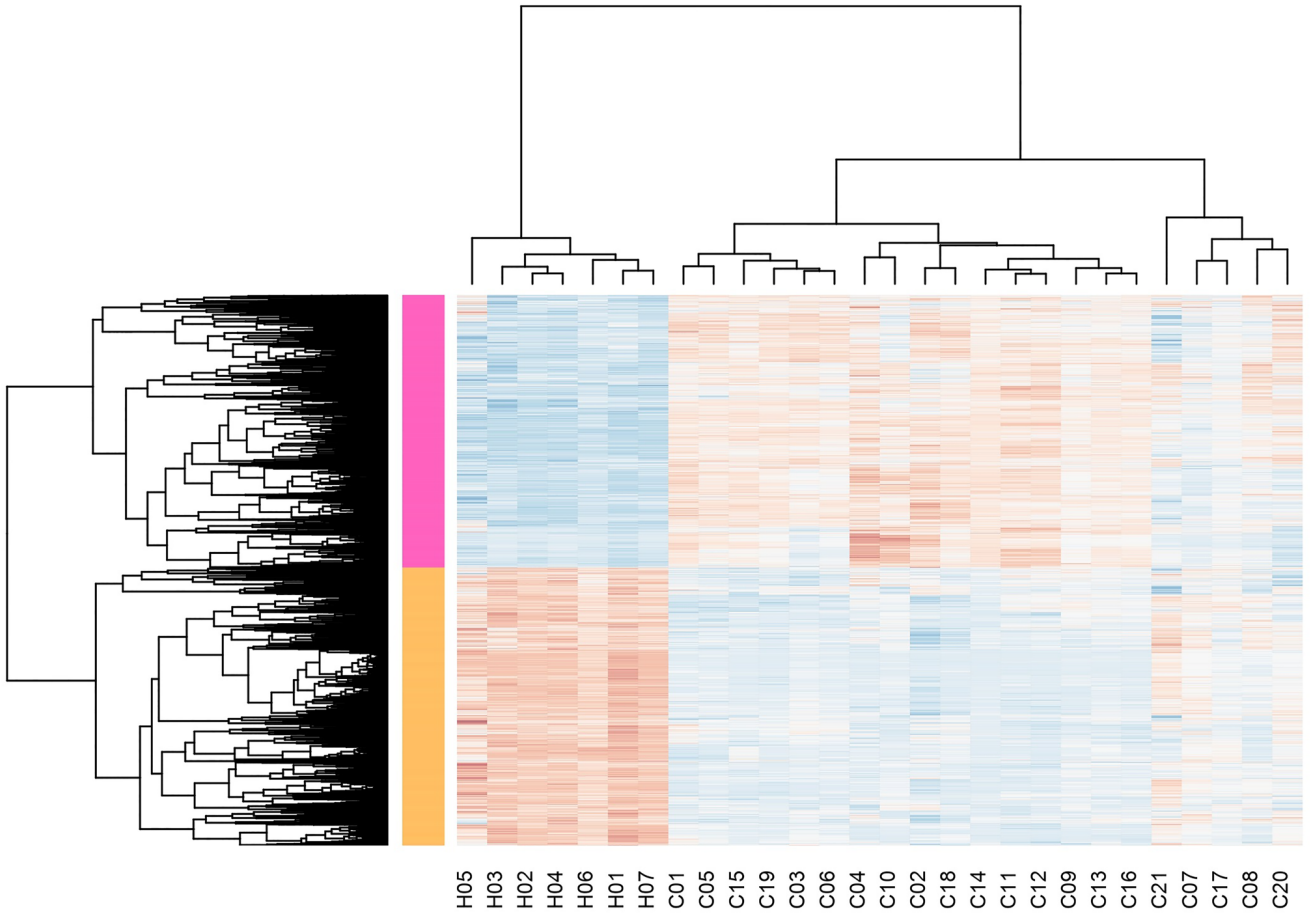
The hierarchical clustering of the genes and samples and a static heatmap of differentially expressed genes (DEGs). This picture shows that there is a clear difference in the expression of the different genes taken from the healthy and diseased samples and that we may reach a meaningful theory by studying them.

Figure 1 shows the hierarchical clustering of the genes and samples and a static heatmap of DGEs. The seven columns on the left are related to the samples of healthy people, and the rest of the columns show the changes in the gene expression of the patients. As can be seen on the heatmap, there is a clear difference in the overall expression of different genes between infected and healthy states, and we will try to get the hidden information.

### Construction of weighted gene coexpression network

To further understand the cause of skin wounds and their molecular mechanisms in CL, we did a weighted gene coexpression network analysis (WGCNA) on the differentially expressed genes (Table S3) to investigate CL from a new perspective in this study. The scattering curve and median of the data for each sample were plotted in Fig. S6. Doing hierarchical clustering on the data matrix (Fig. S7) showed that there were no outliers among the samples.

**Figure 2:**
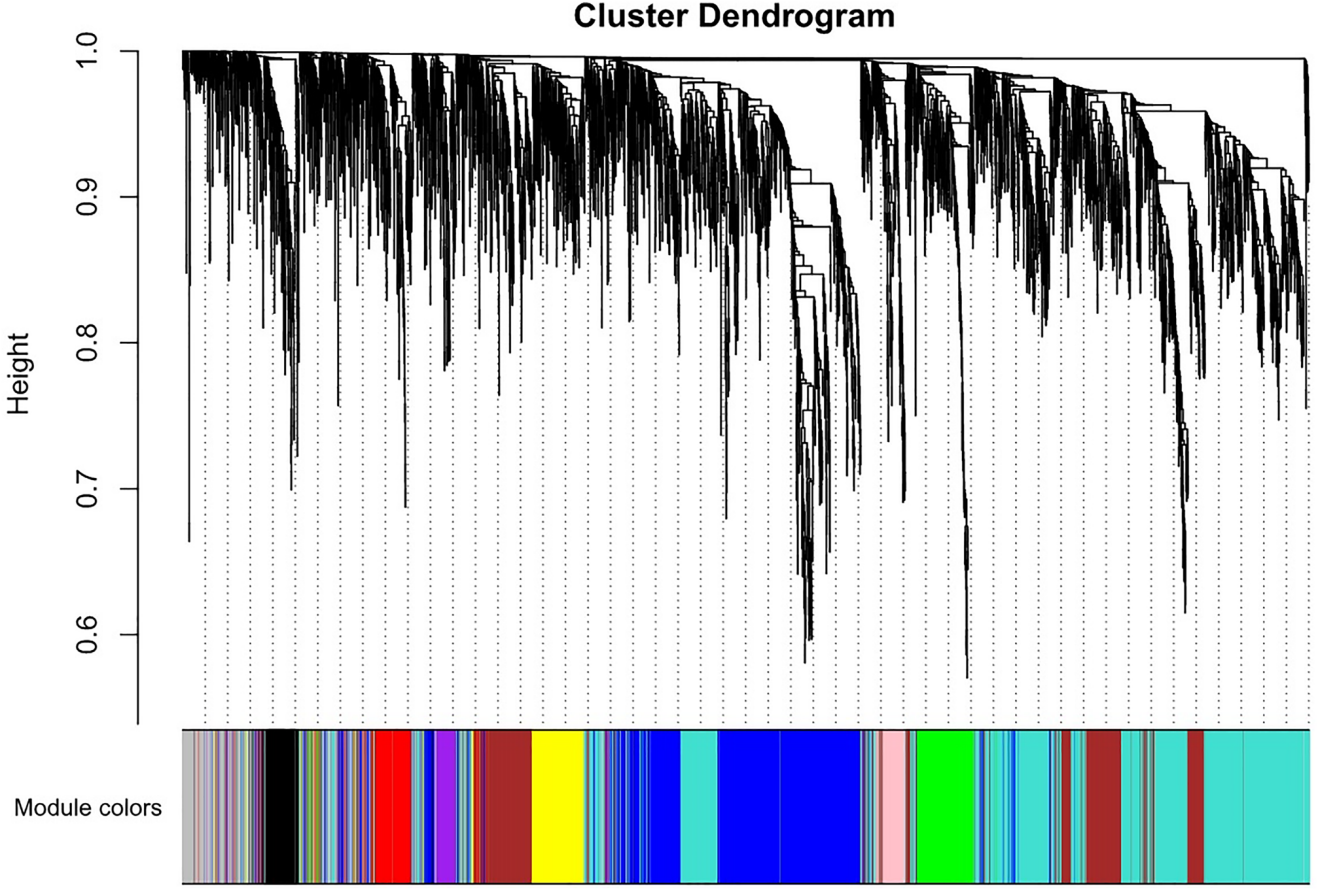
Weighted gene co-expression network (WGCNA) identified several modules, represented by different colors. Modules were grouped with closely linked genes and comparable expression profiles. Then identified on the dendrogram using the Dynamic Tree Cut algorithm. Module identification was accomplished with the dynamic tree-cut method, by hierarchically clustering genes using 1-TOM as the distance measure with mergeCutHeight = 0.25 and minModuleSize = 30 for the resulting dendrogram.

We performed Network topology for thresholding powers from 1 to 20, and the relatively balanced scale independence and mean connectivity of the WGCNA were identified subsequently. An appropriate soft threshold power (soft power = 14) was selected by the standard scale-free networks (Fig. S8). By converting traits to a color representation (white means low, red means high and grey means missing entry) we plotted the sample dendrogram and the colors underneath (Fig. S9). Adjacency was turned into a topological overlap matrix (TOM), which could measure the network connectivity of a gene defined as the sum of its adjacency with all other genes for generating the network (TOMType = "unsigned"), then the corresponding dissimilarity (1-TOM) was calculated. Modules with closely linked genes, and comparable expression profiles, were grouped and then identified on the dendrogram using the Dynamic Tree Cut algorithm. Module identification was accomplished with the dynamic tree-cut method by hierarchically clustering genes using 1-TOM as the distance measure with mergeCutHeight = 0.25 and minModuleSize = 30 for the resulting dendrogram^6^. Several modules were identified and represented by different colors (Fig. 2).

### Relating modules to external clinical traits

**Figure 3:**
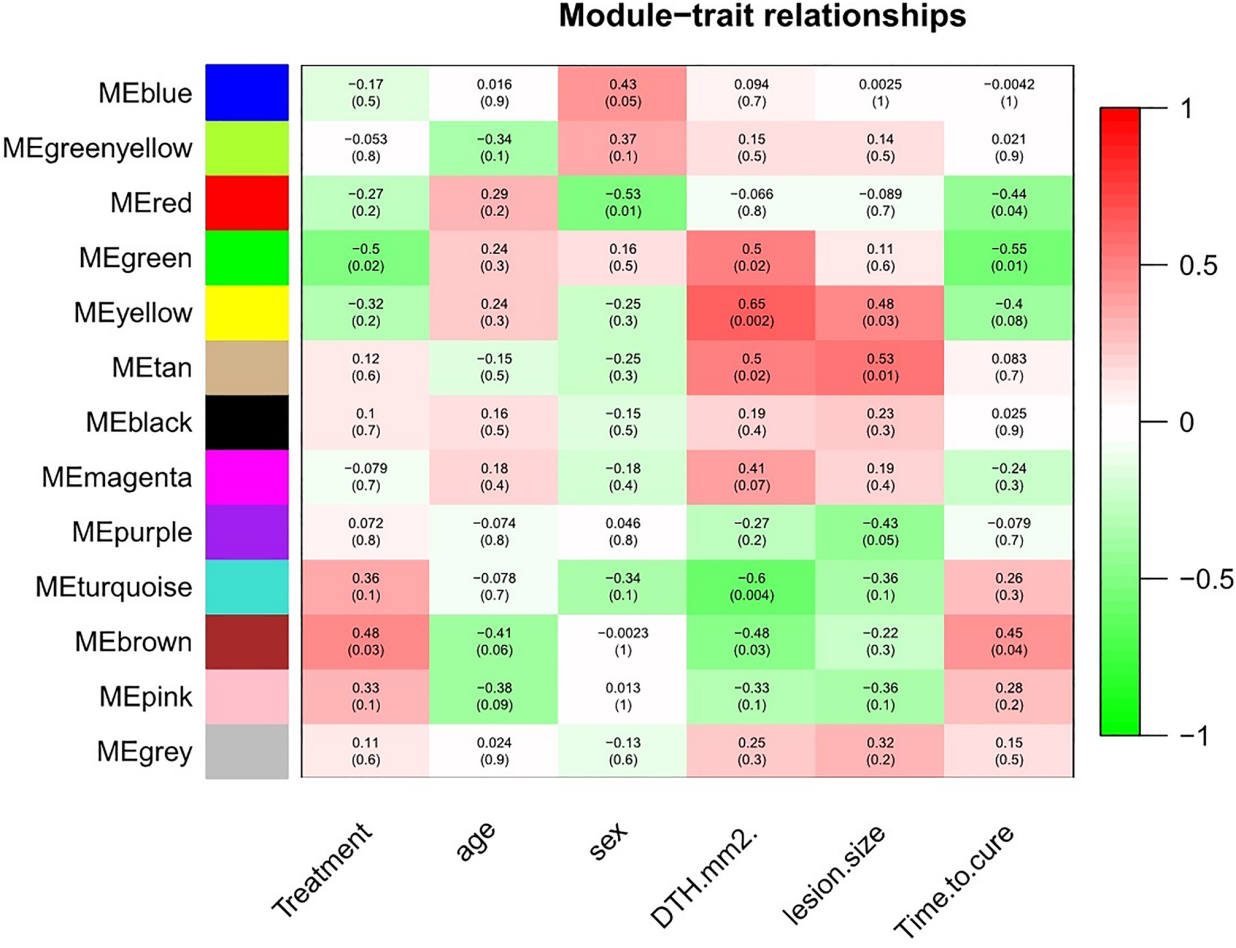
Graphical representation of the results and color-coded each association by the correlation value. Differential expressions of Genes in the "yellow" module are most correlated with the "size" and "DTH" traits (size of cutaneous lesions).

We would like to recognize the modules that are notably associated with the measured clinical traits^16^. Since we are looking for factors that cause skin ulcers, increased hypersensitivity reactions of the skin and the size of the ulcer are the most important factors for the identification of delayed healing wounds of CL. We are looking for genes that are associated with this clinical symptom and display correlations and their p-values. Figure 3 is a graphical representation of the results and color-coded each association by the correlation value.

### Gene relationship to trait and important modules

We measure the correlation between the genes and the traits by defining the Gene Significance (GS). The module membership (MM) was defined as the correlation of the gene expression profile with module eigengenes. Genes, with the highest MM and highest GS in modules of interest, were candidates for further research. Thus, the intra-modular hub genes were chosen by external traits based on GS > 0.2 and MM > 0.6 with a threshold of *P*-value < 0.05.

It can be assumed that the bigger the wound or inflammation area, the greater the skin damage caused by the disease.

We concentrate on "size" and "DTH" as the trait of interest. "Size" indicates the area of the wound caused by CL which is measured in square millimeters. "DTH" (Delayed Type Hypersensitivity) indicates the surface area of inflamed skin after the CL antigen injection. As can be seen in Fig. 3, the "yellow" module is the most correlated with the "size" and "DTH" traits.

We plot a scatterplot of Gene Significance (GS) vs. Module Membership (MM) to the yellow module (R-squared value = cor = 0.34, p = 8.4e−14) (Fig. S10). GS and MM are correlated, indicating that the genes in the yellow module are significantly associated with the grade of wounds caused by CL^17^. Although this correlation is moderate, it is still more significant than other modules. A list of genes in the yellow module is in Table S4.

### Analysis of genes in key module

The list of genes in the yellow module (456 genes) was matched with the list of "Top Hits" (Table S2). The "logFC" and "adjusted p.value" were included in the final list (Table S5). By importing the list and data of the genes in the 'STRING protein query' of Cytoscape software^18^, we could construct a co-expression network of these genes and visualize it as a cluster of 319 genes (Fig. S11). We analyzed this collection with the MCODE application^19^ in Cytoscape software and three gene clusters were identified (Fig. S12, S13, S14). We did a "Functional Enrichment Analysis" on these clusters and the results are summarized in Tables S6, S7, and S8.

### Functional enrichment analysis of the key module and gene clusters

**Figure 4:**
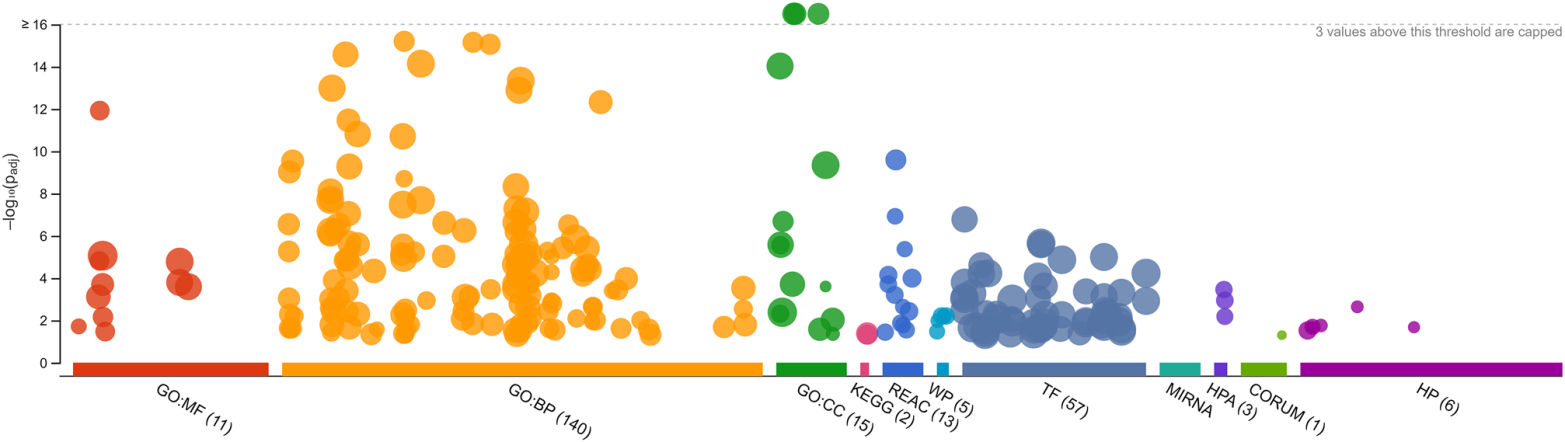
Gene Ontology (GO) enrichment analysis results of the genes in the yellow module (https://biit.cs.ut.ee/gplink/l/BrkqggeNSR) In this module, there are genes from CC (Cellular Component), BP (Biological Process) and MF (Molecular Function) groups with a significantly increased expression.

For the Gene Ontology (GO) enrichment analysis of the key module and its clusters, we used the website of "g:profiler"^20^. Figure 4 shows a summary of the GO enrichment analysis results of the genes in the yellow module and the detailed results are in this link (https://biit.cs.ut.ee/gplink/l/BrkqggeNSR).

Figures S15, S16, and S17 show the results of the GO analysis of the selected gene clusters from the yellow module in the previous section. The most significant results have been summarized in Table S9.

### Validating the results

To validate our results, we did a DGE analysis on GSE63931^21^ and GSE55664^22^ datasets. The results in both cases are similar to our training data set. The full results of DGEs are shown in Tables S10 and S11. We gathered the DGEs results of clusters one to three in Table S12. All the genes that were isolated in the previous stages and placed in three different clusters have significant and similar expression changes in the new databases and can cause the same complications.

### Deconvolution of RNA-seq data

The goal of transcriptome deconvolution is to estimate an RNA sample's cellular composition from its gene expression data, which can then be used to correctly identify sample-to-sample composition differences. The measured values in bulk transcriptomic data are an average of gene expression across all cell types in heterogeneous samples made up of multiple cell types. By Deconvolution methods, we can estimate cell type fractions in bulk RNA-seq data. In recent years, several deconvolution techniques have been published, most of them using 'cell type-specific gene expression references'^23,24^.

To further investigate the immunological changes in the skin tissue affected by Leishmania, we used the "granulator" package of the R program^25^ to estimate immunologic cell type proportions based on the available gene expression data. This can be used in the subsequent analysis of the cell-type heterogeneity. The bulk expression profiles of mixed tissue samples and the 'reference data' are inputs of the deconvolution method to estimate the abundance of cell types in each sample^26,27^.

We used "sigMatrix_ABIS_S0—PBMCs reference profile—(17 cell types)" as the reference profile. This is bulk RNA-seq gene expression data of peripheral blood mononuclear cells (PBMCs) from 12 healthy donors^28^. The file is publicly available on the NCBI database under the GEO accession number GSE107011^29^. We plot the cell-type similarity matrix of all reference profiles by computing their Kendall Rank Correlation^30^ (Fig. S18). The figure shows the similarity of gene expression profiles in 17 immunological cells, which are considered reference profiles. All T cells and NK cells have almost similar gene expression profiles. B cells and plasma cells are in the next group, and mononuclear cells are in a separate group. According to these results, we can trust the accuracy of this reference profile in the correct diagnosis of immunological cells.

**Figure 5:**
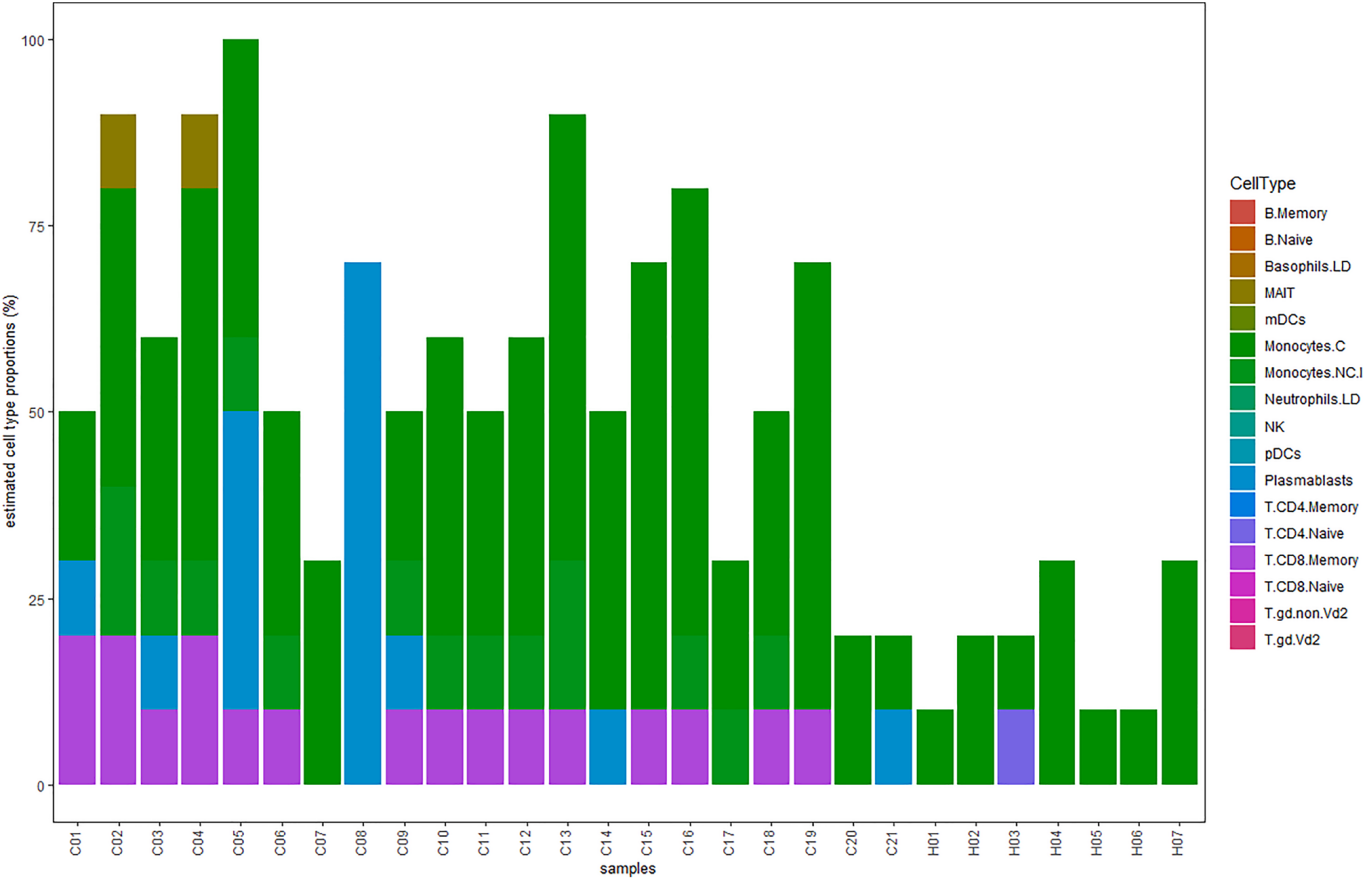
Percentage of immunologic cells. The cell type proportions computed by the "Support Vector Regression model (SVR)" using the sigMatrix_ABIS_S0 reference profile. The percentage of immunologic cells has increased significantly in the tissues infected with parasites. Most of them are Monocytes and T Cells which can play an important role in tissue cytotoxic activities.

When reasonable reference profiles have been produced, we can utilize that to estimate cell type extends from the tissue mass RNA-seq dataset. Figure 5 shows the cell type proportions computed by the "Support Vector Regression model (SVR)" using the sigMatrix_ABIS_S0 reference profile.

As shown in Fig. 5, in healthy samples, the number of immunologic cells is only about 20%, but the percentage of these cells has increased significantly in the tissues infected with parasites. Most of them are Monocytes and T Cells which can play an important role in tissue cytotoxic activities.

### Ethical approval

This article does not contain any studies with human participants or animals performed by any authors. In this paper, I present the results of the second chapter of my doctoral dissertation, titled "Bioinformatics Analysis of differential gene expression in cutaneous leishmaniasis Lesions" with Research Ethical Committee Certificate: IR.IAU.TABRIZ.REC.1401.179.

## Discussion

Patients with CL-caused skin ulcers continue to receive inadequate treatment^31^. The underlying pathophysiological mechanisms of these wounds are complicated. WGCNA can provide valuable insights into complex genetic networks by mining valuable data.

We performed co-expression network analyses, on RNAseq data taken from 21 skin biopsies of CL and 7 healthy samples. There were 6,096 differentially expressed genes, with adj.P.value > 0.05 and LogFC > 0.8. By doing WGCNA on these data, the module of genes whose expression changes had the greatest effect on causing skin ulcers was determined (Table S5). These 456 genes were the proposed causative biomarkers of skin ulcers in CL. To limit their number, we grouped these genes based on Functional analysis, and three important operational groups were identified among them, whose works were related to wound healing.

The genes in the first group (Fig. S12) are *IFIT1, DDX58, SAMD9, ISG15, HERC6, DDX60, IFI6, IFI44L, USP18, OAS1, ISG20, IFI44, OAS3, PARP9, IFIT3*, and *MX1* (Tables S6 and S9). Most of these genes are responsible for the production of cytokine, interferon alpha/beta signaling, regulators of DDX58/IFIH1 signaling, and antiviral defense. The unregulated production of type I IFN can be harmful, resulting in chronic cellular toxicity and inflammatory diseases^32^. Uncontrolled expression changes of these genes can lead to tissue damage. Although all these genes have significant expression changes, *OAS1* is the most notable gene in this group and can be assumed as a hub gene.

The second group (Fig. S13) includes *COL3A1, COL6A3, MMP2, LOXL1, LAMB1, VCAN, TIMP3, CDH11, FN1, SERPINH1, PLOD1, LAMA2, TGFB3, COL5A2, ADAMTS2* and *COL5A1* (Tables S7 and S9). Most of them are involved in collagen-containing extracellular matrix organization and collagen formation.

Collagen is a key component of the extracellular matrix and plays a crucial role in regulating the phases of the wound's healing, either in its native fibrillary conformation or as soluble components in the wound environment. Impairments in any of these phases leave the wound in a chronic, non-healing state that typically requires some intervention to complete the process. During normal wound healing, collagen acts as a scaffold for cell entry and growth in the wound bed and promotes new collagen deposition. The role of collagen in wound healing is to attract fibroblasts and promote the deposition of new collagen in the wound bed^33^. It has been reported that failures in the production of collagen, can be seen in most wounds that are slow to heal^33,34^. In this group, *SERPINH1* has the most significant relationship to other genes and can be assumed as a hub gene. The encoded protein is localized to the endoplasmic reticulum and plays a role in collagen biosynthesis as a collagen-specific molecular chaperone^35^.

The third group (Fig. S14) includes *MFAP4, MFAP4, BMP1, EFEMP1, PPARG, NID2, RUNX2, MFAP2, FBLN1, TGFBR1, TLL1, FBLN5, CD34* and *ELN* (Table S8 and S9). Most of them are involved in Extra Cellular Matrix (ECM) organization, collagen and elastic fiber formation, and matrix metalloproteinase.

All mammalian tissues are composed of the ECM, a network primarily composed of collagen, elastin, and their associated microfibrils, fibronectin, and laminins embedded in a viscoelastic gel of anionic proteoglycan polymers. The cellular microenvironment supports many functions in addition to its structural role, it promotes cell proliferation, adhesion, and migration, and regulates cell differentiation and death^36^. Several non-collagenous proteins are found in the ECM, each with a specific binding site for other matrix macromolecules and receptors on the cell's surface. Consequently, these proteins contribute both to matrix organization and cell attachment^37^. A change in gene expression of this group can disrupt ECM activity and fail to heal of the wounds. *FBLN1* has the most significant relationship with other genes in this group and can be assumed as a hub gene.

## Conclusions

The differential Gene Expression analysis shows numerous alterations in the gene expression of cutaneous leishmaniasis lesions and normal skin. The most important expression changes are seen in genes that are responsible for: (1) producing tissue-damaging cytokines (2) production and activation of collagen and fibrin proteins (3) disrupting the extracellular matrix. All of these, cause skin wounds and/or prevent their healing. This data can furnish better approaches to managing the undesirable and hurtful impacts of leishmaniasis.

## Supplementary Information

Supplementary Information 1.

Supplementary Information 2.

Supplementary Information 3.

Supplementary Information 4.

Supplementary Information 5.

Supplementary Information 6.

Supplementary Information 7.

Supplementary Information 8.

Supplementary Information 9.

Supplementary Information 10.

Supplementary Information 11.

Supplementary Information 12.

Supplementary Information 13.

Supplementary Information 14.

Supplementary Information 15.

Supplementary Information 16.

Supplementary Information 17.

Supplementary Information 18.

Supplementary Information 19.

Supplementary Information 20.

Supplementary Information 21.

Supplementary Information 22.

Supplementary Information 23.

Supplementary Information 24.

Supplementary Information 25.

Supplementary Information 26.

Supplementary Information 27.

Supplementary Information 28.

Supplementary Information 29.

Supplementary Information 30.

## Data Availability

The datasets generated and/or analyzed during the current study are available in the Gene Expression Omnibus database, https://www.ncbi.nlm.nih.gov/geo/ repository.
