# Supplementary figures and images for: Identification of molecular mechanisms causing skin lesions of cutaneous leishmaniasis using weighted gene coexpression network analysis (WGCNA)
Source: Sci Rep. 2023 Jun 17;13:9836. doi: 10.1038/s41598-023-35868-0 (PMC10276835; doi:10.1038/s41598-023-35868-0)

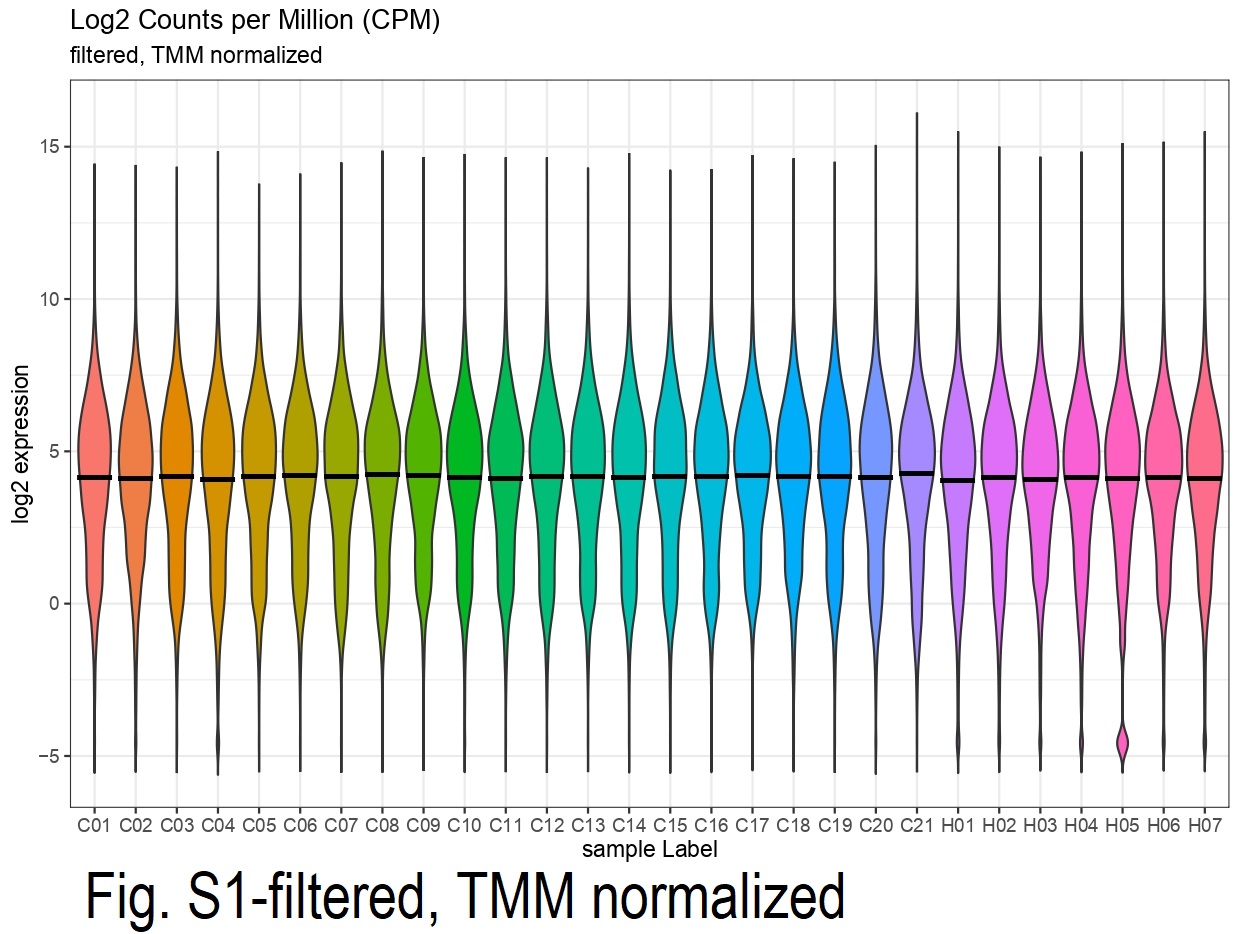

Supplement: Supplementary file 1 — Supplementary Information 1. [file 41598_2023_35868_MOESM1_ESM.jpg]

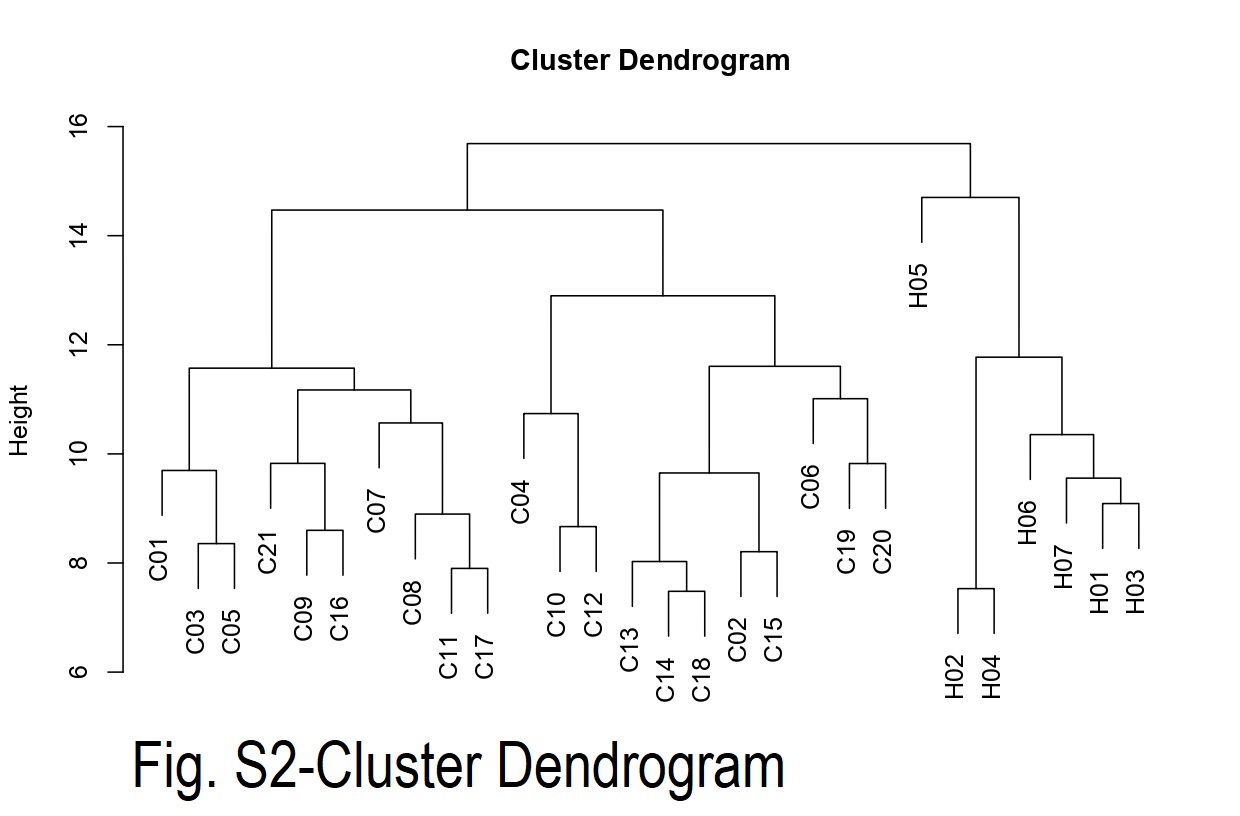

Supplement: Supplementary file 2 — Supplementary Information 2. [file 41598_2023_35868_MOESM2_ESM.jpg]

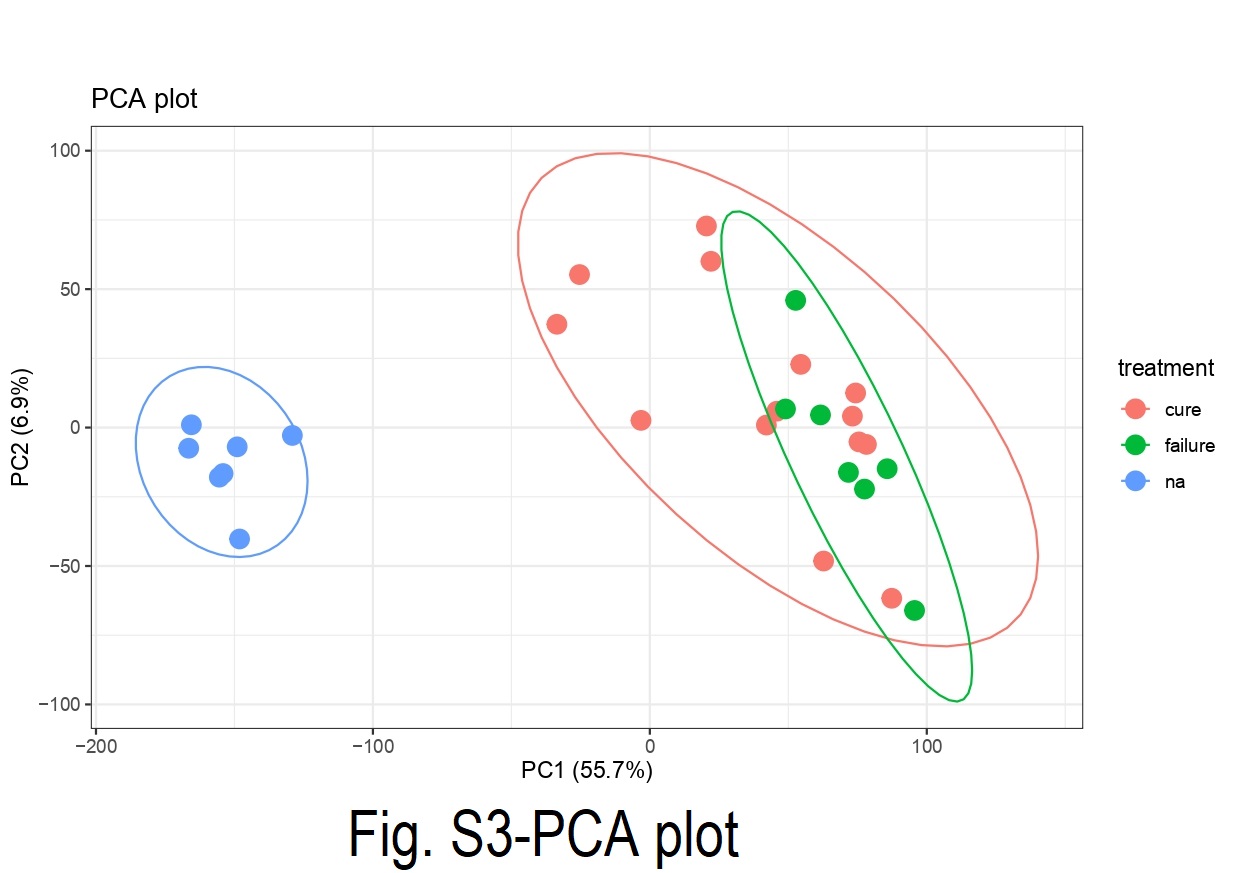

Supplement: Supplementary file 3 — Supplementary Information 3. [file 41598_2023_35868_MOESM3_ESM.jpg]

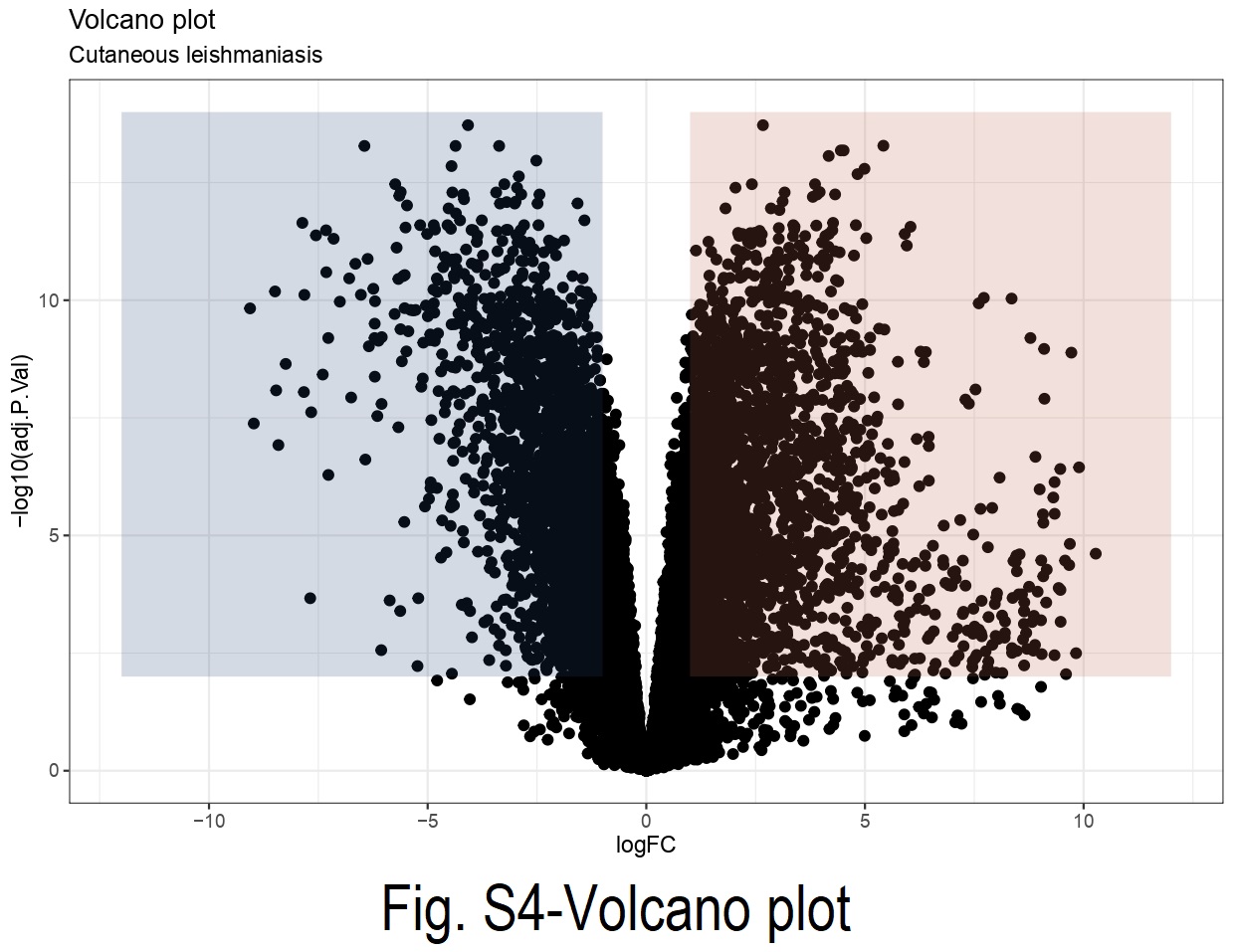

Supplement: Supplementary file 4 — Supplementary Information 4. [file 41598_2023_35868_MOESM4_ESM.jpg]

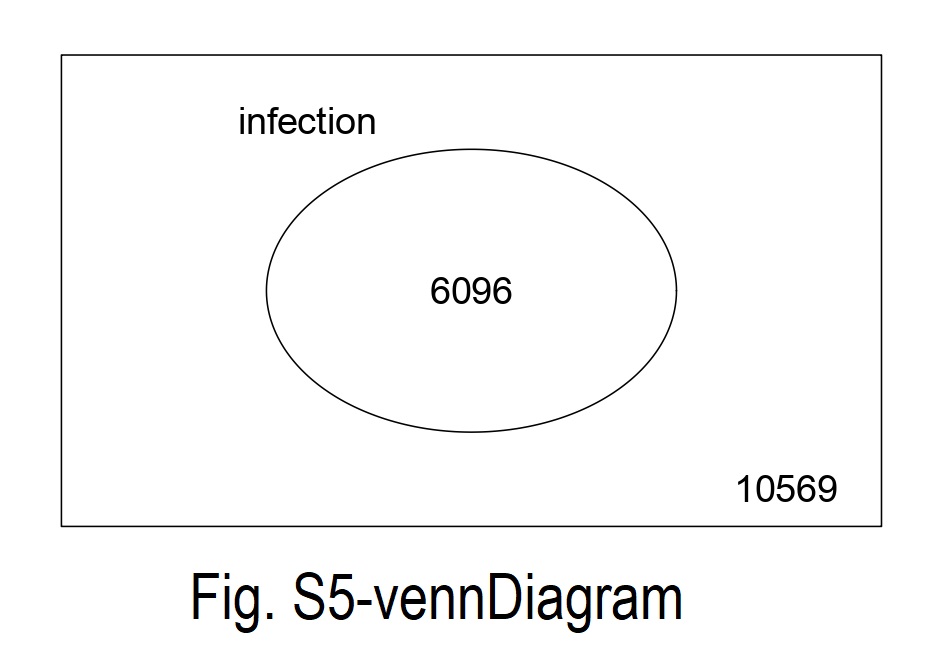

Supplement: Supplementary file 5 — Supplementary Information 5. [file 41598_2023_35868_MOESM5_ESM.jpg]

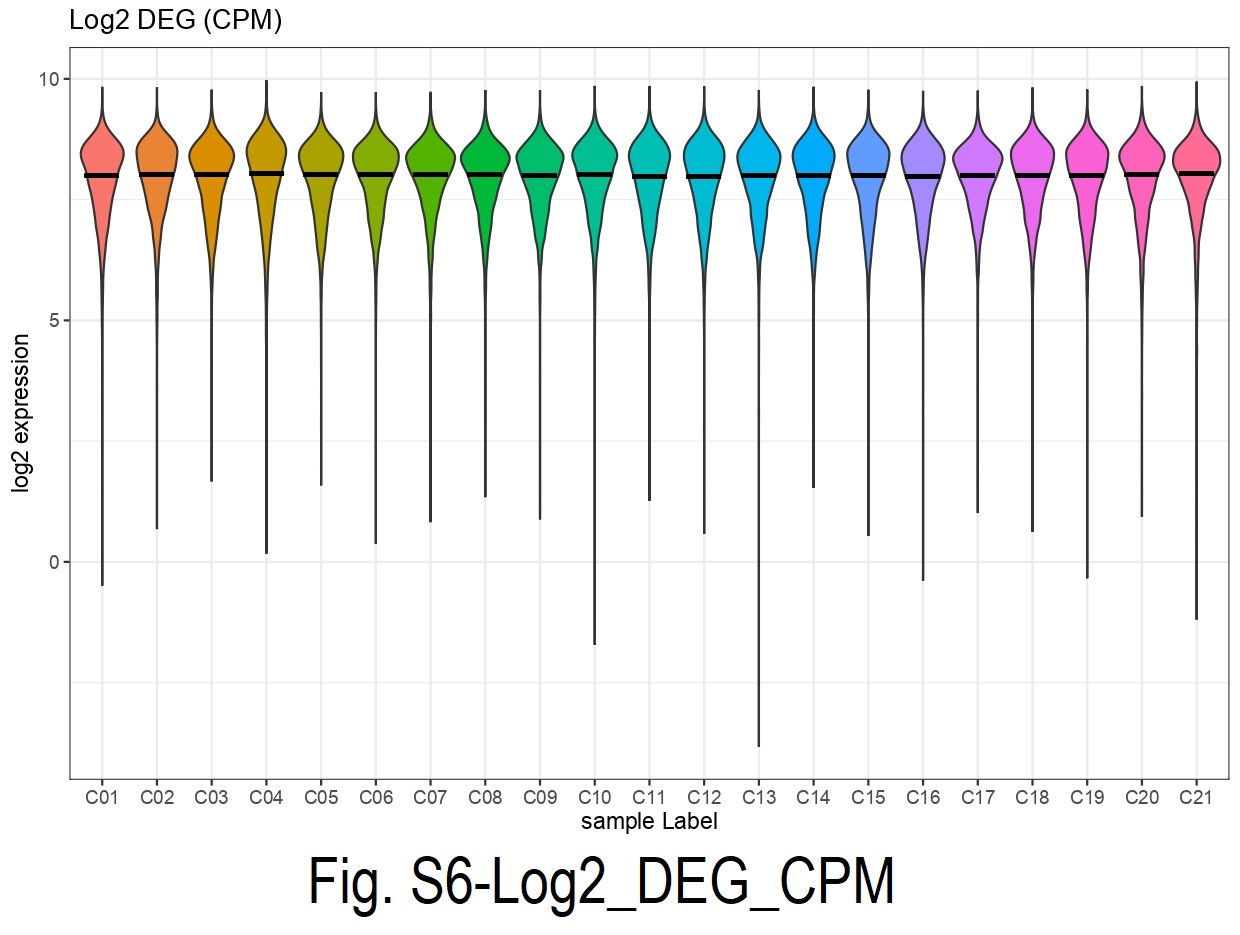

Supplement: Supplementary file 6 — Supplementary Information 6. [file 41598_2023_35868_MOESM6_ESM.jpg]

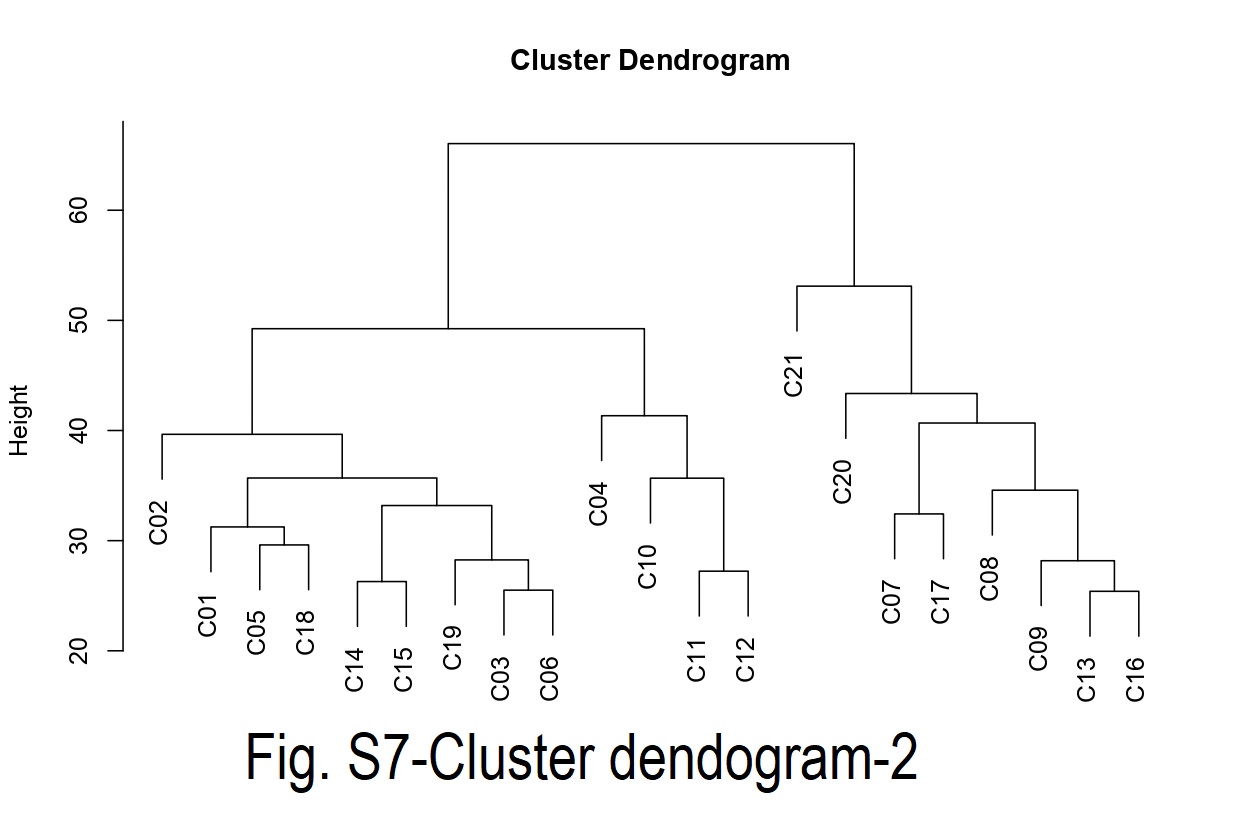

Supplement: Supplementary file 7 — Supplementary Information 7. [file 41598_2023_35868_MOESM7_ESM.jpg]

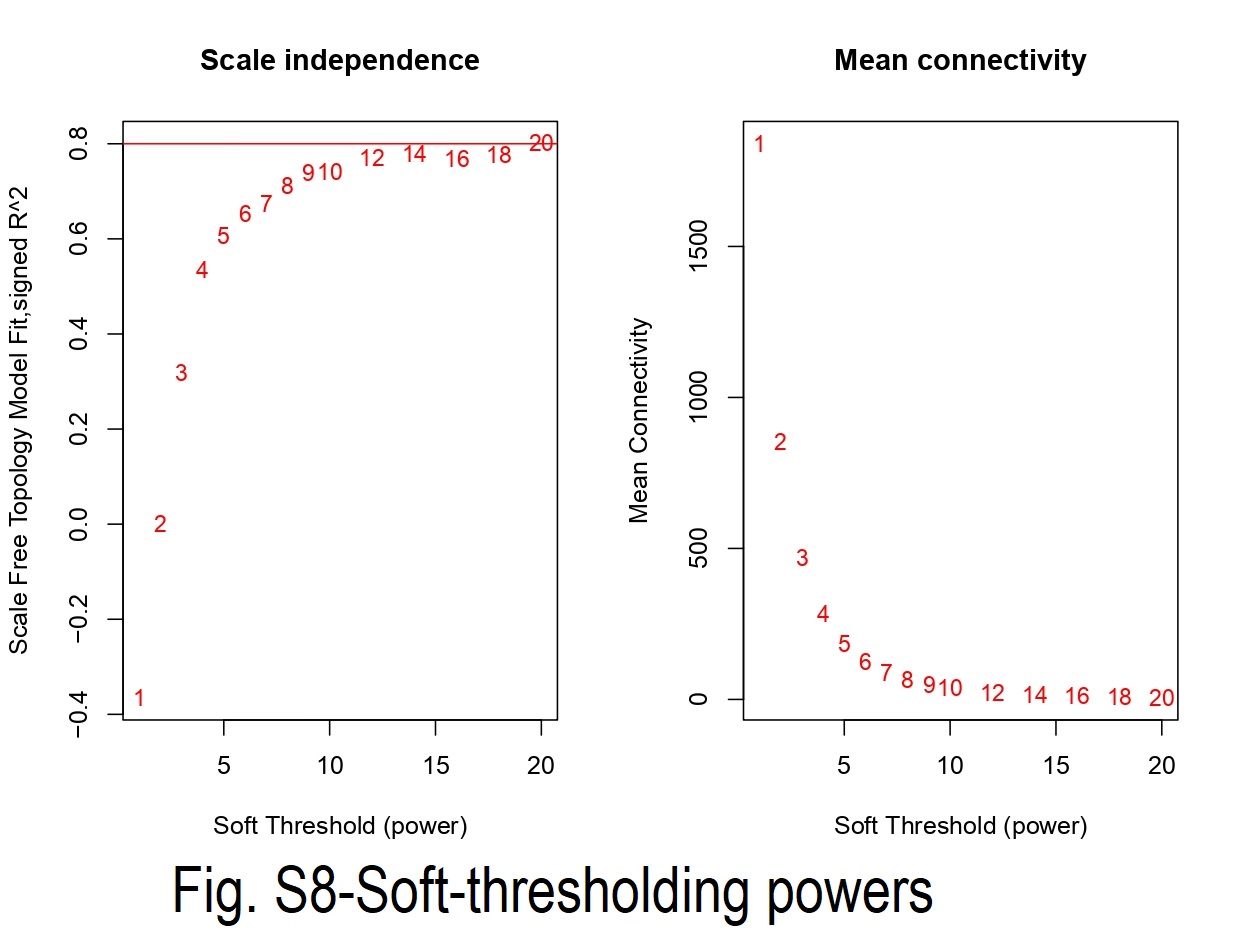

Supplement: Supplementary file 8 — Supplementary Information 8. [file 41598_2023_35868_MOESM8_ESM.jpg]

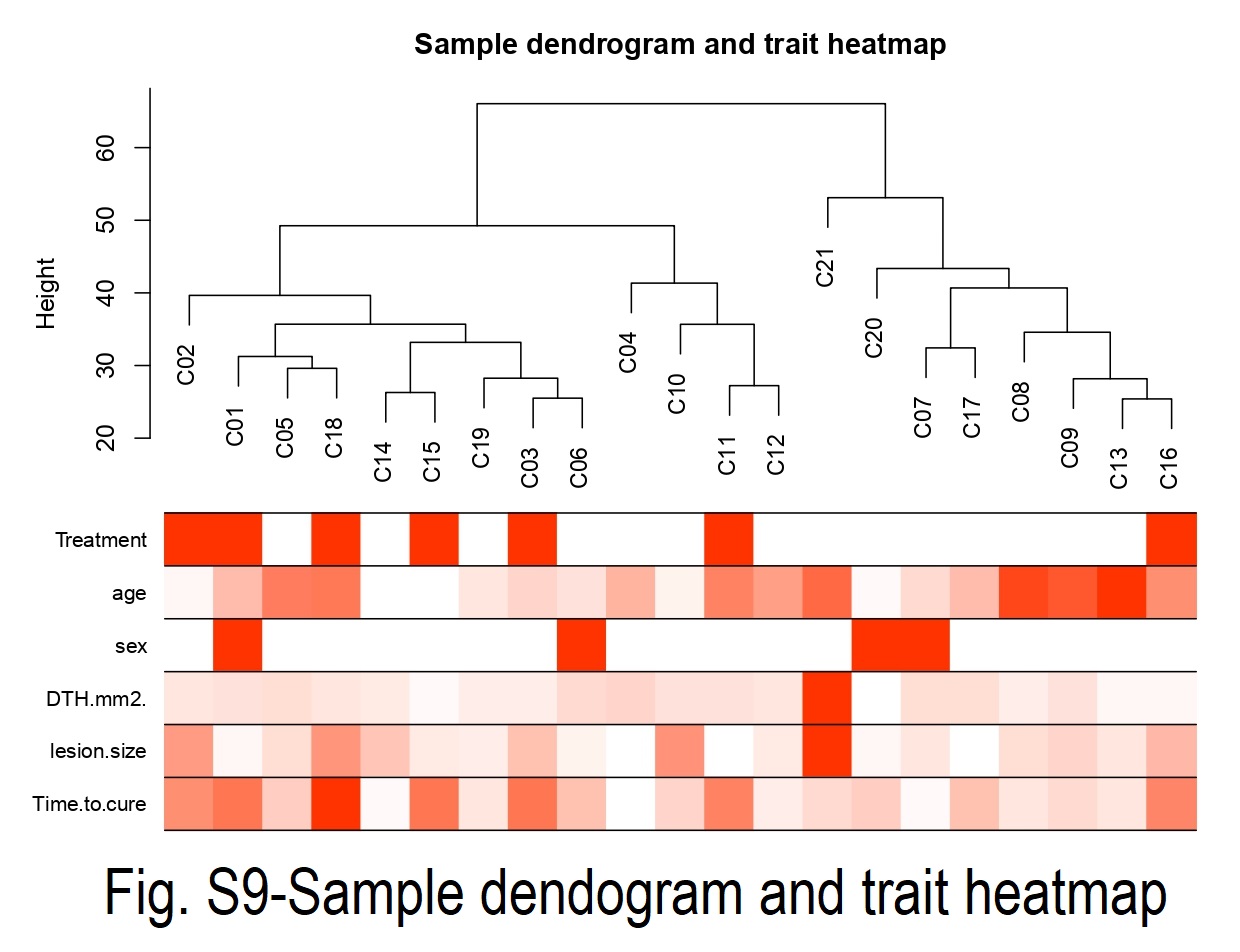

Supplement: Supplementary file 9 — Supplementary Information 9. [file 41598_2023_35868_MOESM9_ESM.jpg]

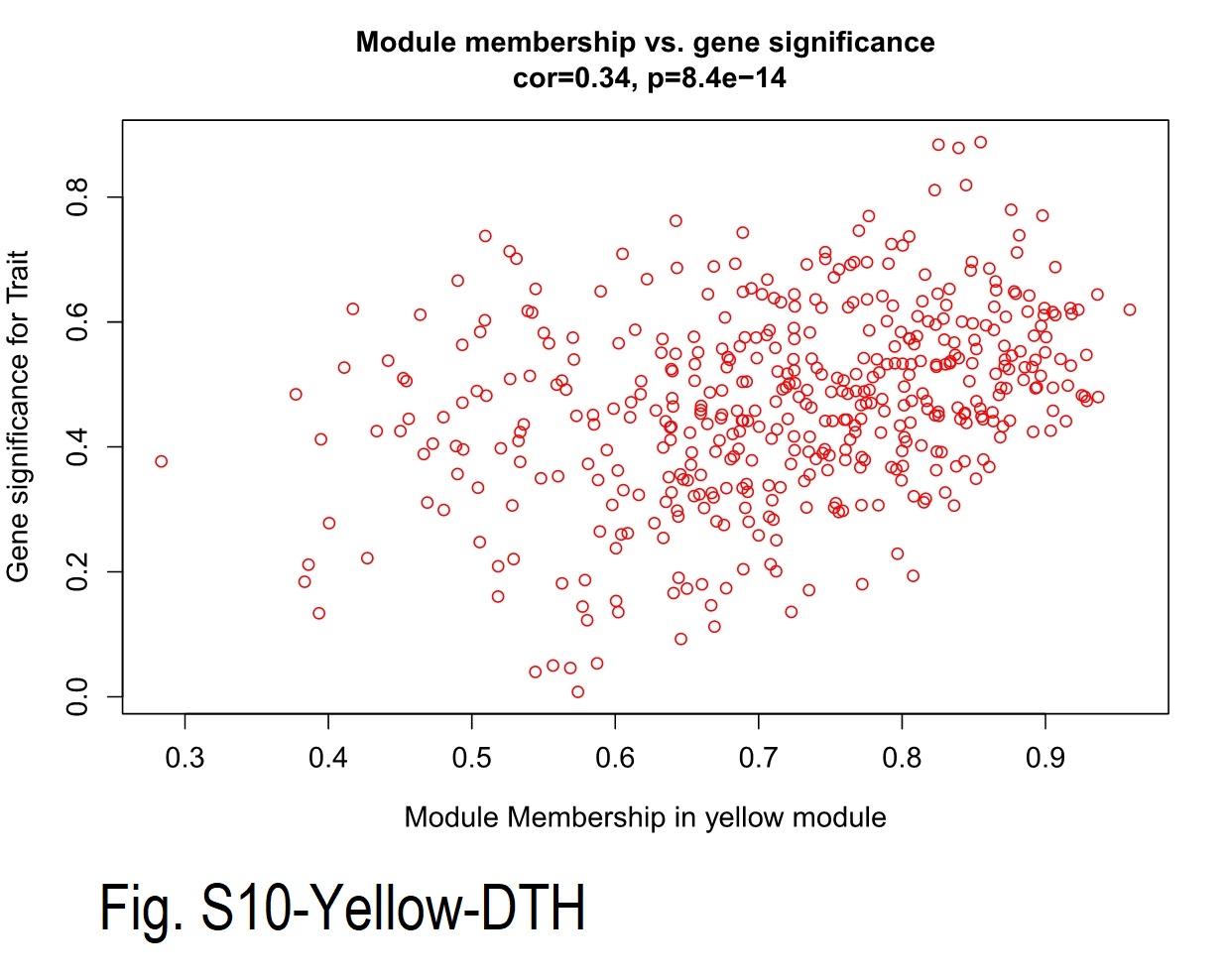

Supplement: Supplementary file 10 — Supplementary Information 10. [file 41598_2023_35868_MOESM10_ESM.jpg]

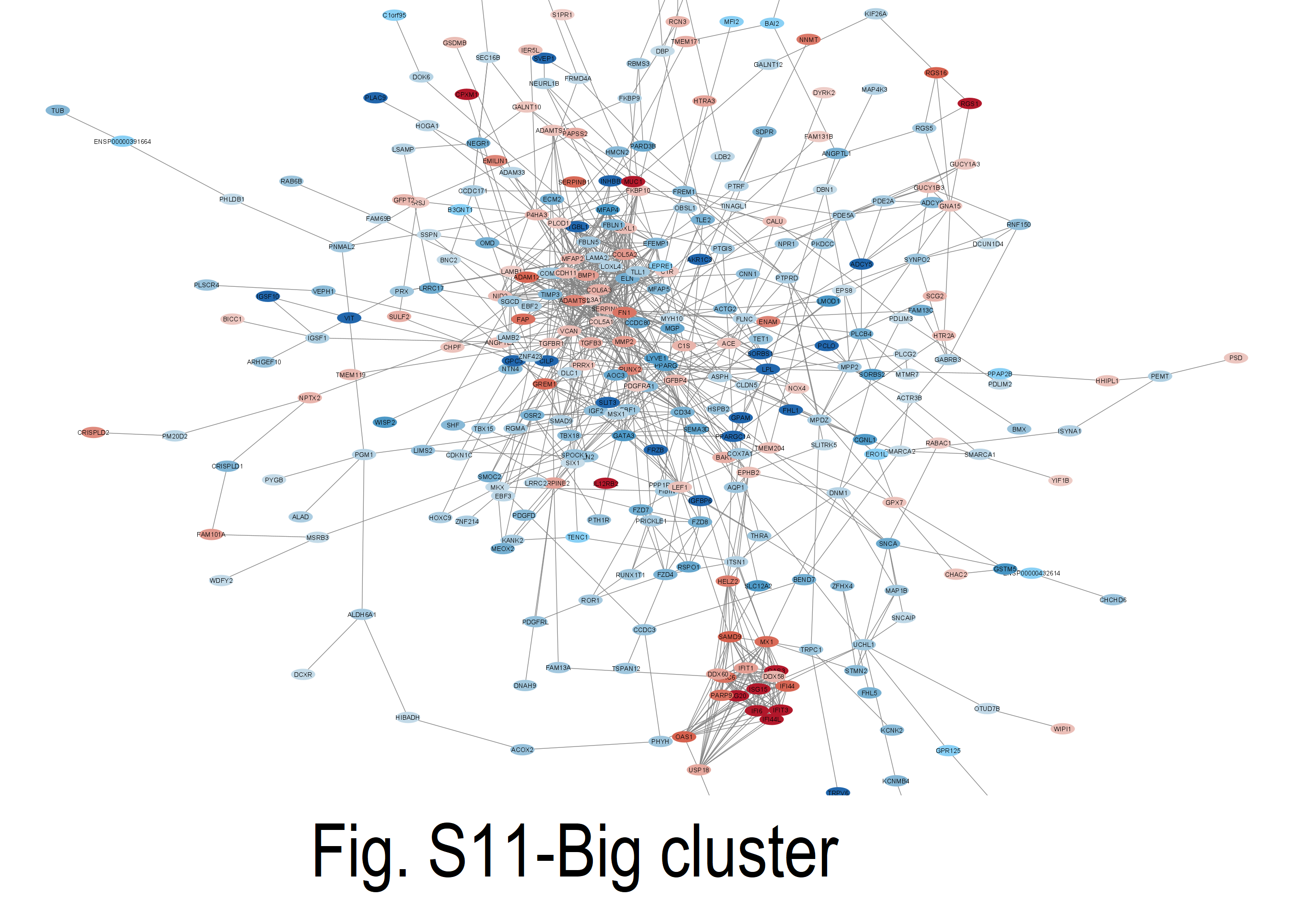

Supplement: Supplementary file 11 — Supplementary Information 11. [file 41598_2023_35868_MOESM11_ESM.png]

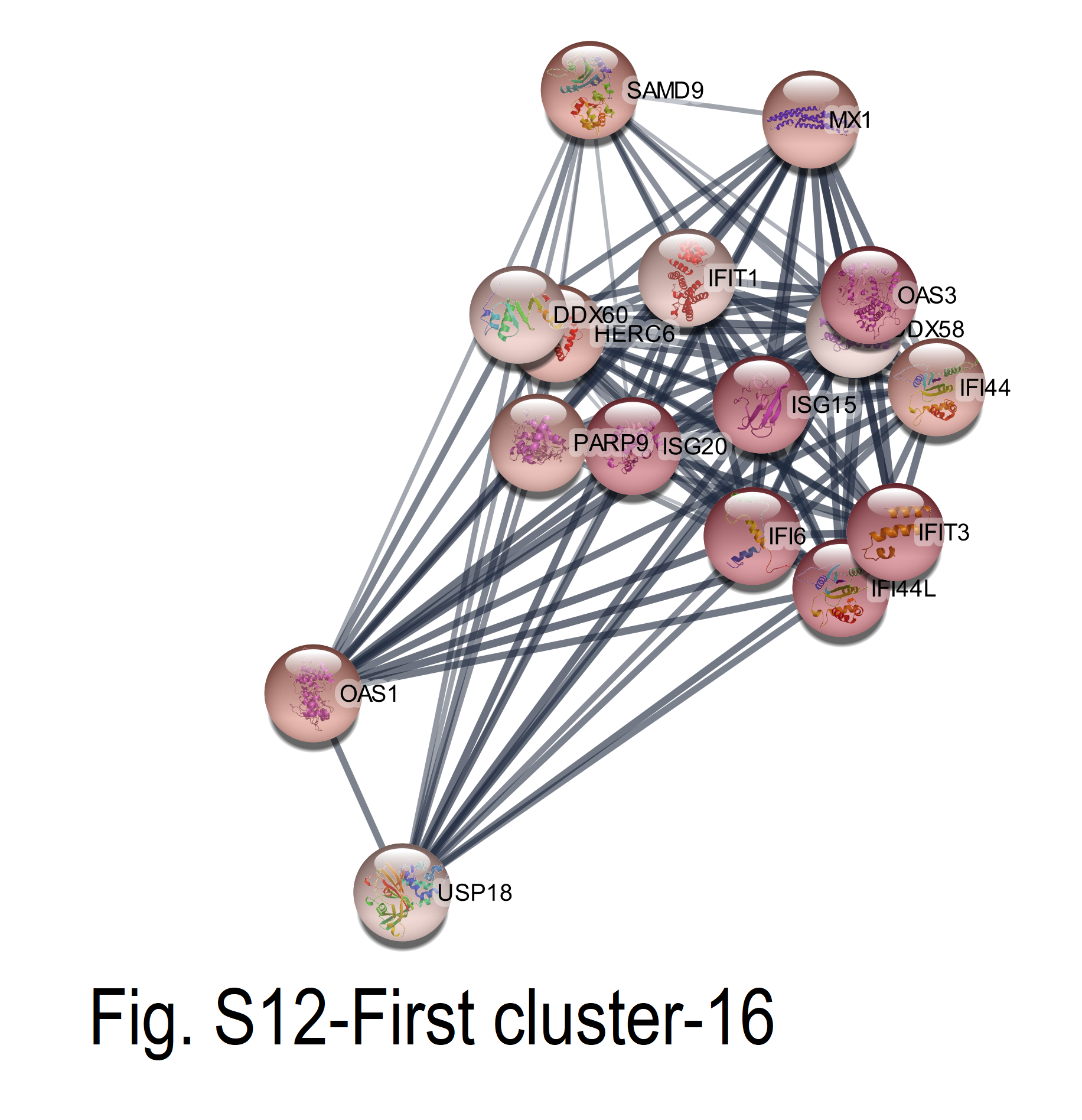

Supplement: Supplementary file 12 — Supplementary Information 12. [file 41598_2023_35868_MOESM12_ESM.png]

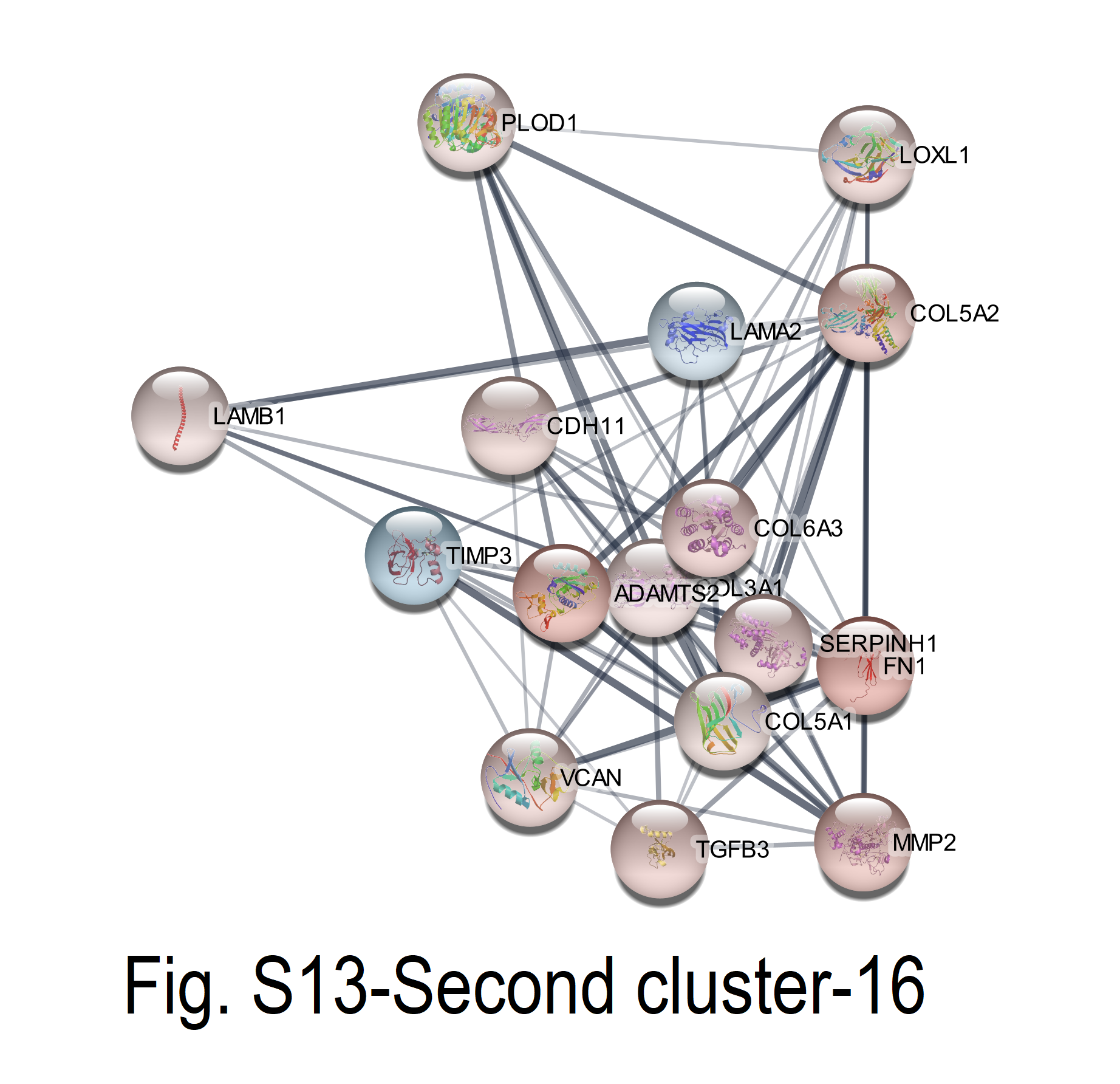

Supplement: Supplementary file 13 — Supplementary Information 13. [file 41598_2023_35868_MOESM13_ESM.png]

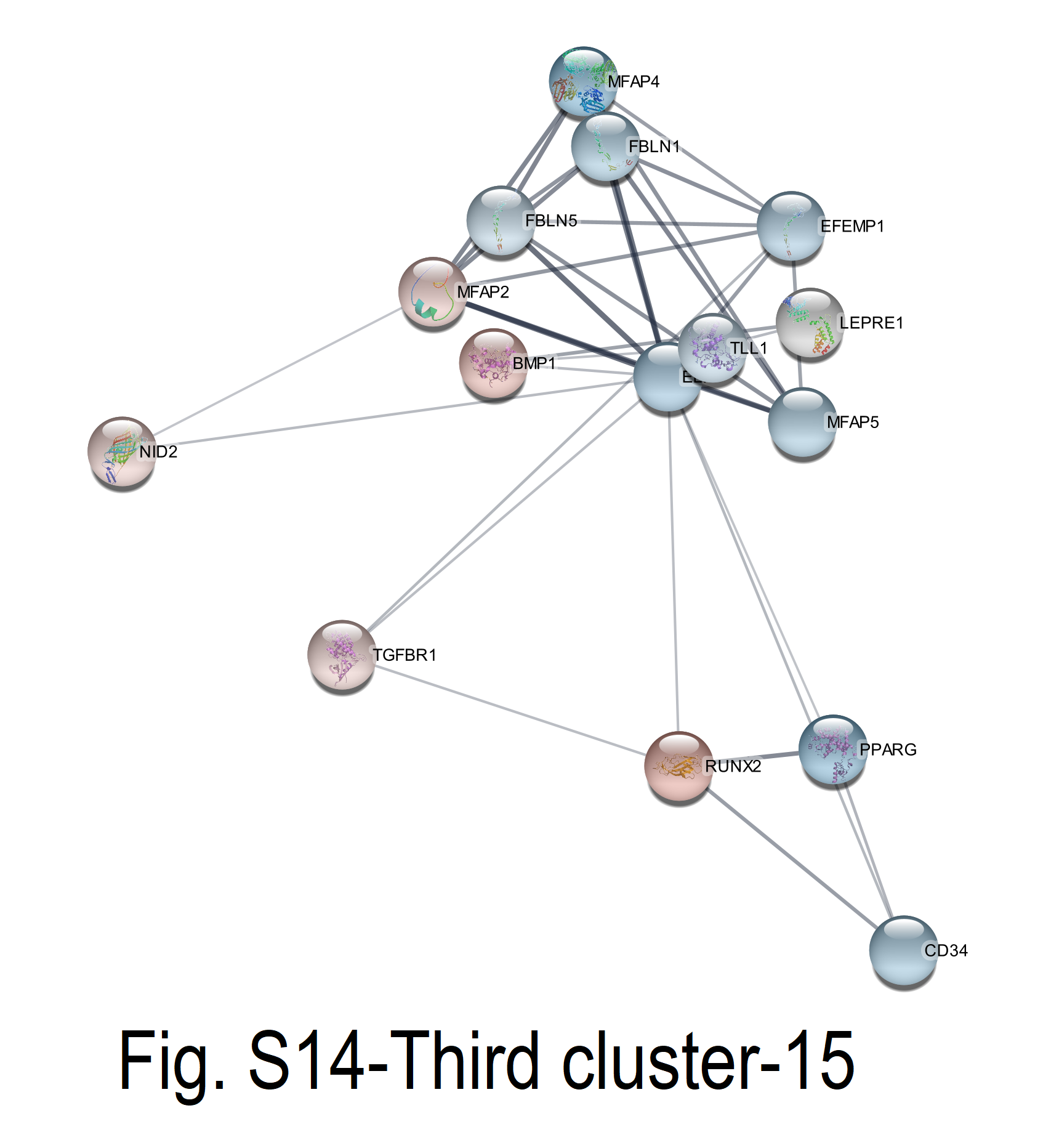

Supplement: Supplementary file 14 — Supplementary Information 14. [file 41598_2023_35868_MOESM14_ESM.png]

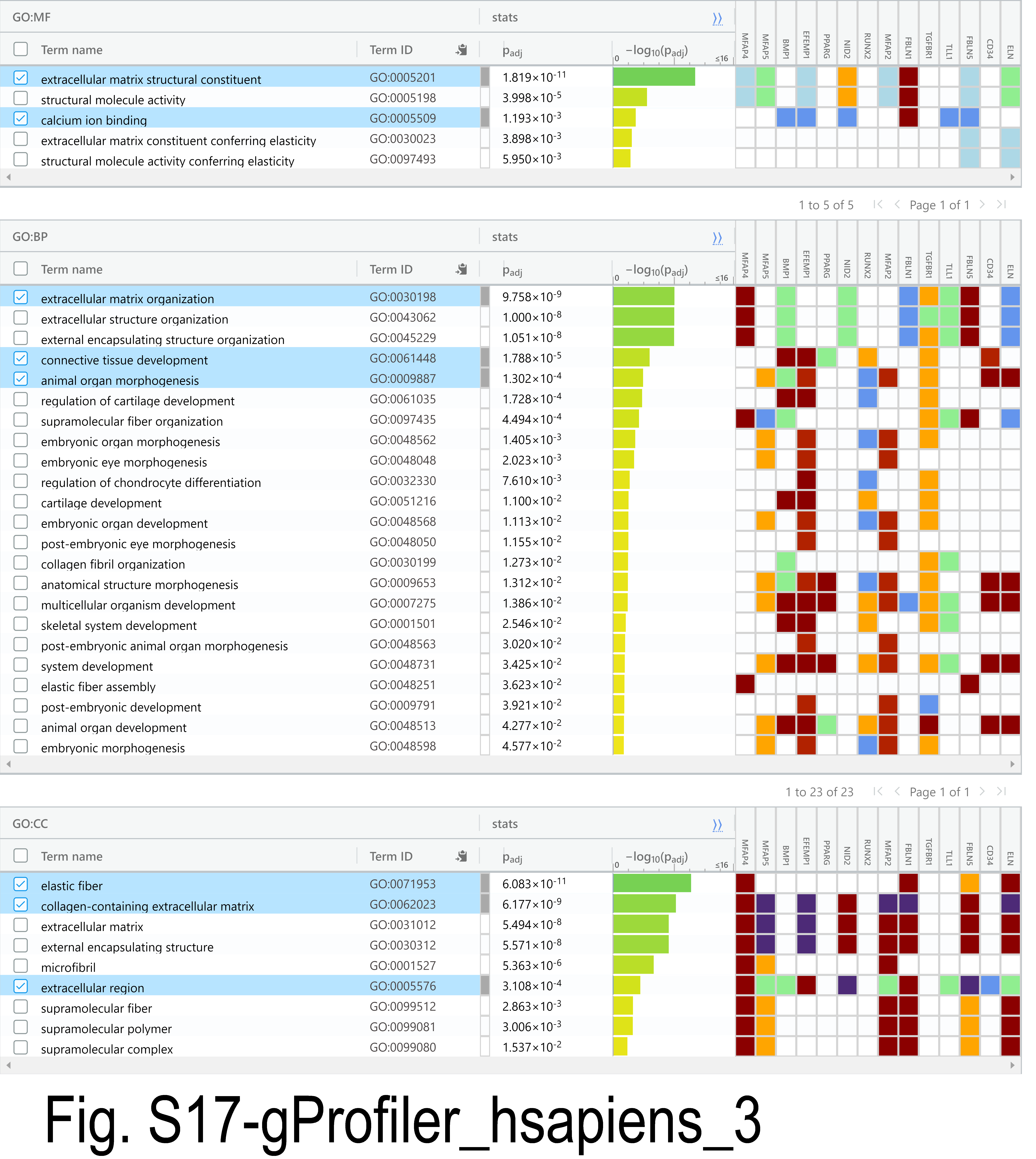

Supplement: Supplementary file 17 — Supplementary Information 17. [file 41598_2023_35868_MOESM17_ESM.png]

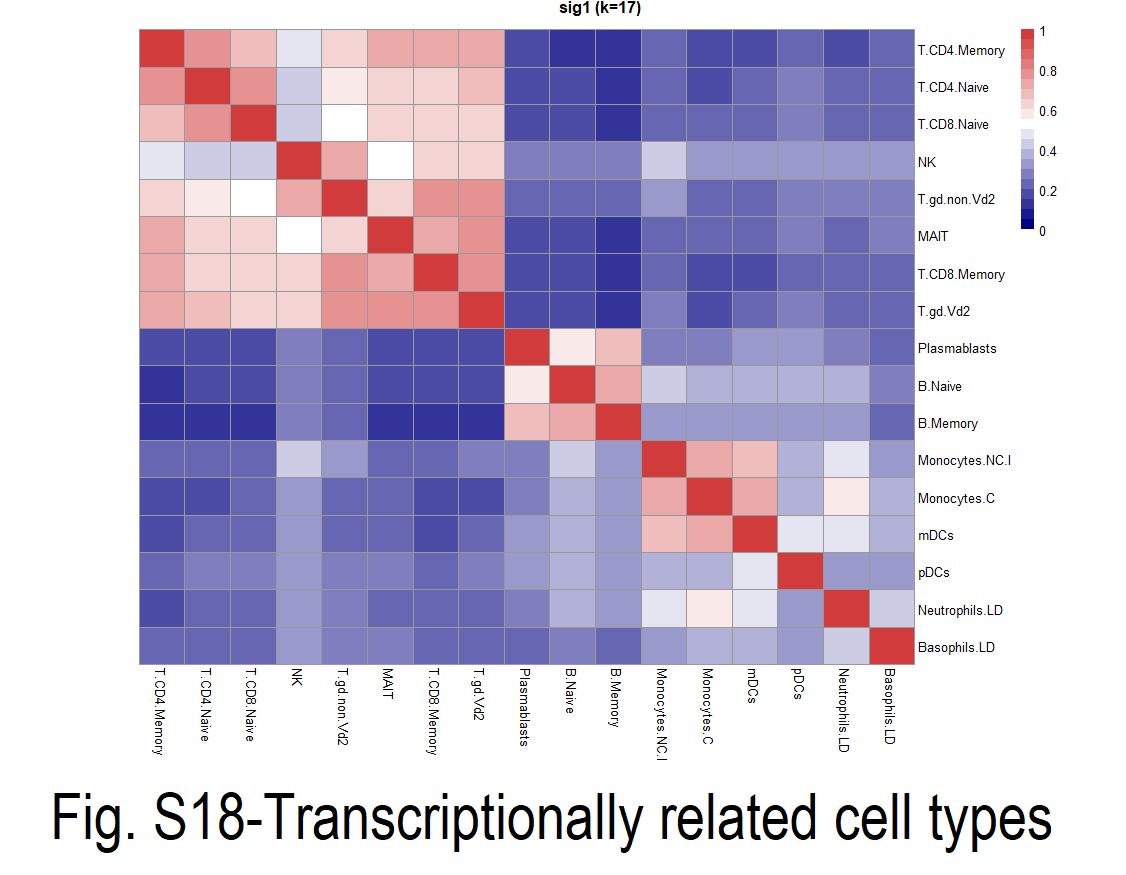

Supplement: Supplementary file 18 — Supplementary Information 18. [file 41598_2023_35868_MOESM18_ESM.png]
